# Algorithm Optimization in Methylation Detection with Multiple RT-qPCR

**DOI:** 10.1371/journal.pone.0163333

**Published:** 2016-11-29

**Authors:** Lele Song, Yuemin Li, Jia Jia, Guangpeng Zhou, Jianming Wang, Qian Kang, Peng Jin, Jianqiu Sheng, Guoxiang Cai, Sanjun Cai, Xiaoliang Han

**Affiliations:** 1 The Chinese PLA 309th hospital, Beijing, People’s Republic of China; 2 BioChain (Beijing) Science and Technology, Inc, Beijing, People’s Republic of China; 3 Department of Gastroenterology, The Army General Hospital, Beijing, People’s Republic of China; 4 Department of Colorectal Surgery, Fudan University Shanghai Cancer Center, Shanghai, People’s Republic of China; Queen's University Belfast, UNITED KINGDOM

## Abstract

Epigenetic markers based on differential methylation of DNA sequences are used in cancer screening and diagnostics. Detection of abnormal methylation at specific loci by real-time quantitative polymerase chain reaction (RT-qPCR) has been developed to enable high-throughput cancer screening. For tests that combine the results of multiple PCR replicates into a single reportable result, both individual PCR cutoff and weighting of the individual PCR result are essential to test outcome. In this opportunistic screening study, we tested samples from 1133 patients using the triplicate Epi proColon assay with various algorithms and compared it with the newly developed single replicate SensiColon assay that measures methylation status of the same *SEPT9* gene sequence. The Epi proColon test approved by the US FDA (1/3 algorithm) showed the highest sensitivity (82.4%) at a lower specificity (82.0%) compared with the Epi proColon 2.0 CE version with 2/3 algorithm (75.1% sensitivity, 97.1% specificity) or 1/1 algorithm (71.3% sensitivity, 92.7% specificity). No significant difference in performance was found between the Epi proColon 2.0 CE and the SensiColon assays. The choice of algorithm must depend on specific test usage, including screening and early detection. These considerations allow one to choose the optimal algorithm to maximize the test performance. We hope this study can help to optimize the methylation detection in cancer screening and early detection.

## Introduction

Worldwide, colorectal cancer (CRC) is the third most common malignancy in men and the second in women [1]. Regular screening and early detection of CRC can achieve effective prevention. However, 60%-70% of CRC patients are not diagnosed until they are symptomatic at later stages, and only 11.8% of cases are detected at early stages [2]. It is therefore urgent to reduce the CRC morbidity and mortality by improving participation in screening. Participation in screening using current methods, including colonoscopy and fecal blood testing, varies widely, and there are significant barriers posed by the methods that limit their use. Recently, the introduction of blood based screening provides an additional option that may increase screening rate.

The plasma-based *SEPT9* gene methylation assay, developed as the Epi proColon test, was recently approved by the US FDA as the first blood-based CRC screening test. It has been shown to be effective for the early detection and screening of CRC, supported by a number of case-control and prospective screening studies [3–6]. The test pre-analytics are designed to extract cell free DNA (cfDNA) from a 3.5 mL plasma sample, perform bisulfite conversion and purify bisulfite converted DNA (bisDNA). The PCR assay measures *SEPT9* methylation in triplicate PCR reactions using the bisDNA derived from the 3.5 ml plasma sample. This is based on the consideration that abnormally methylated cfDNA occurs at a very low concentration in the blood, potentially in the single digit copies per milliliter, in the background of much higher concentration of normal genomic DNA. In order to distinguish the low copy number aberrantly methylated DNA from background, the test needs to be very sensitive while maintaining sufficient specificity.

Data interpretation in multiple PCR diagnosis poses a challenge, as positive interpretation from a single PCR from a set of replicate reactions may generate high specificity at the price of reducing sensitivity, while positive interpretation requiring more than one reaction from a set of replicates would result in higher sensitivity at the cost of reducing specificity. Optimizing sensitivity and specificity to achieve the best performance of an assay for its intended use is a key step in developing a diagnostic product, as is observed in Reciever Operator Characteristic (ROC) analysis where an optimized Area Under the Curve (AUC) is derived based on the relationship of these two parameters. As an example, given that the Epi proColon test is run in triplicate, interpretation can be adjusted by requiring only one, two or all three replicates to be positive for the result to be determined positive, and these differences shift the sensitivity / specificity performance of the test.

The choice of algorithm is dependent on the purpose of a test. If tests aim at excluding as many negative subjects as possible, such as those for early detection purpose, high specificity should be prioritized and positive interpretation from more than one PCR replicates should be considered. In contrast, if tests aim at detecting as many positive subjects as possible, such as those for disease screening, high sensitivity is the priority and positive interpretation from one PCR should be considered. The choice of algorithm is also dependent on the rules of different healthcare systems. Most healthcare systems favor high specificity tests in screening to avoid expensive follow-up procedures, but high sensitivity is favored in order to avoid missing cancers in the US system. In the US, the Epi proColon algorithm requires only one positive, emphasizing the highest sensitivity. In Europe, the Epi proColon 2.0 CE algorithm requires at least two positive results, placing a greater emphasis on test specificity. It is clear that the assay exhibited distinct sensitivity and specificity when different algorithms were applied, and clear and thorough analysis of same set of data side-by-side should be performed to illustrate and scientifically prove the impact of 1/3 and 2/3 algorithm. This would avoid the confusion in understanding the fluctuation of Epi proColon test results in different publication. This is one purpose of this study because the choice of algorithm will impact the power of the test and indication for application.

In this study, we performed opportunistic screening using the CE-marked Epi proColon 2.0 CE assay. Opportunistic screening has been proven as an effective way to screen in the hospital environment [7]. It occurs when potential patients come to their doctors for a health examination or test due to illness or discomfort. Doctors use this opportunity to encourage these patients to attend a disease screening program. We analyzed the four possible algorithms: 1/3, 2/3, 1/1 and 3/3, and the new SensiColon assay, a single replicate *SEPT9* assay recently approved by the Chinese FDA (CFDA) [7], in matched patients. Distinct sensitivity and specificity were calculated using various algorithms and compared in order to identify the optimal algorithm for opportunistic screening. Our results show that the single PCR *SEPT9* assay is equally effective as the 2/3 algorithm Epi proColon 2.0 CE assay in opportunistic screening. This new single PCR *SEPT9* assay simplifies the test procedure, lowers the test costs with no compromise in test performance, and therefore may exhibit higher compliance.

## Materials and Methods

### Ethics

The plan for the trial was submitted to the ethics committee of the participating hospitals for review and approval before the start of the clinical trial. All subjects signed the informed consent before blood or stool collection, and they were informed of the usage of plasma and the test results. Confirmation of approval for clinical trials or studies was received from all named institutional review board or ethics committee. The participating institutions and the members of review board or ethics committee are listed below:

The Chinese PLA 309^th^ Hospital: Liqin Wang, Nan Ye, Haotian Yu, Wenqiao Wang, Chen Yao, Yue Zheng, Nan Li, Guokun Ao, Yumei Liang, Guanren Zhao, Hongqun Cheng, Hong Wang, Xia Shen

The Army General Hospital: Ying Han, Shushan Shi, Xiaojun Peng, Xingyou Wang, Xiaohui Di, Yingxin Chang, Baisuo Xu, and Lin Xu.

Fudan University Cancer Hospital: Jiong Wu, Chaosu Hu, Yingqiang Shi, Huaying Wang, Zhenqi Lu, Jiliang Yin, Ye Guo, Zhiqiang Meng, Weijun Peng, Ji Zhu, Qing Zhai, Quanxing Ni, Shaogang Yang, Yueqin Diao, Qin Lu, and Weijing Zhang.

### Study design, patients, and colonoscopy

The opportunistic screening study was designed and implemented in three Chinese hospitals using the Epi proColon 2.0 CE assay. Clinical status was not determined before blood draw for *SEPT9* assay, and blood samples were obtained from all subjects who met the selection criteria. All technicians were blinded to the clinical information of subjects. A total of 1133 subjects were recruited in this study, including 369 CRC patients, 113 subjects with advanced adenoma, 87 subjects with polyps, 27 subjects with inflammatory bowel diseases (IBD), 47 subjects with other GI diseases (ulcer, colitis, etc) and 490 subjects with no evidence of disease (NED) (Table 1). Here polyps refer to inflammatory polyps or hyperplastic polyps, and adenoma refers to adenomatous polyps. The classification of all conditions was based on diagnosis from colonoscopy and subsequent pathological examinations. Subjects with systemic inflammatory, malabsorptive diseases, acute medical conditions, and other malignant diseases were excluded before grouping.

**Table 1:** The number of enrolled subjects by diagnostic groups.

Diagnosis group	Description	Total	Gender		Age			
			Male	Female	<50	50–59	60–69	≥70
CRC	Overall	369	192	177	63	107	108	91
	Stage 0	21	10	11	2	10	5	4
	Stage I	42	20	22	9	11	13	9
	Stage II	105	59	46	14	26	32	33
	Stage III	131	62	69	22	40	43	26
	Stage IV	15	8	7	3	5	5	2
	Not Specified	55	33	22	13	15	10	17
Adenoma		113	74	39	28	35	32	18
Polyps		87	62	25	34	34	10	9
IBD		27	14	13	16	8	1	2
other GI diseases		47	27	20	23	18	4	2
NED		490	260	230	256	133	71	30
Total		1133	629	504	420	335	226	152

CRC = colorectal cancer, IBD = inflammatory bowel disease, NED = no evidence of diseases

CRC patients were stratified by the anatomic appearance of the tumor and then characterized by histopathology. They were divided into six subgroups based on the cancer stage. Patients with incomplete stage information were grouped into ‘Not Specified’. All 1133 subjects underwent a blood draw before colonoscopy and subsequent biopsies or surgery was performed. None of the patients with cancer received chemotherapy, radiotherapy, or surgical intervention before the blood draw and colonoscopy.

### Sample size estimation

Sample size estimation was based on the following equation for known positive detection rate: N = Z^2^*[p (1-p)]/E^2^. The parameters were defined as follows: Z is a statistical parameter (Z = 1.96 for 95% CI); E represented the error (5% was chosen in this study), and P represented the probability of a positive (putative positive detection rate). The p value (0.68) was selected from existing literature for *SEPT9* sensitivity in screening [5,6]. From this, an estimated 334 CRC cases were required. To account for potential incomplete information, tracking, loss of samples, etc. From the estimation that CRC accounts for 30% of high-risk outpatients at least 1113 patients should be included; therefore, the study goal was to recruit 1336 patients, anticipating a 20% loss of follow-up rate (Table 1).

### Sample collection and storage

Samples were collected from outpatients or inpatients, and the sample information was recorded in sample collection forms. A 10-ml peripheral blood sample was collected with 10-ml K_2_EDTA anticoagulant tubes to ensure the accuracy of the assay. Sample storage and transportation followed the instructions for use of the Epi proColon 2.0 assay.

### DNA extraction and qualitative PCR analysis of SEPT9

DNA extraction from plasma samples and bisulfite conversion were performed according to the manufacturer’s instructions of Epi proColon 2.0 CE test (Epigenomics AG, Berlin, Germany). The bisDNA was assayed with Epi proColon 2.0 CE on an AB7500 Fast Dx Real Time PCR device (Life Technologies). PCR was performed in triplicate with 15 μL template DNA per well and run for 45 cycles. PCR results for Beta-actin (ACTB) and methylated *SEPT9* for each of the triplicate reactions were recorded using the instrument software. The validity of each sample batch was determined on the basis of methylated *SEPT9* and ACTB threshold count (Ct) values for the positive and negative controls. ACTB was used as an internal reference to assess the integrity of each sample. The assay procedure for the SensiColon assay was detailed in previous studies [7].

### Data analysis using various algorithm

The data from the PCR reactions of the Epi proColon 2.0 CE assay was analyzed using four algorithms, including the 1/3, 2/3, 1/1 or 3/3 algorithm. 1/3 algorithm means that a sample was considered to be positive if at least one of the three PCRs were positive and was considered to be negative if all three PCR replicates were negative. The 2/3 algorithm means that a sample was considered to be positive if at least two of the three PCRs were positive and was considered to be negative if at least two PCRs were negative. The 3/3 algorithm means that a sample was considered to be positive if all three PCRs were positive and was considered to be negative if at least one PCR was negative. Statistics on Epi proColon 2.0 assay by 1/1 algorithm was performed by calculating the mean values of sensitivity and specificity from the three individual PCR reactions. As the newly developed *SEPT9* SensiColon assay performed only one PCR reaction, the positive or negative result was confirmed from the single PCR. Statistical analysis was performed and the ROC curves were plotted with Graphpad Prism 5.0 software (GraphPad Software, Inc, La Jolla, CA 92037, USA).

## Results

### Data interpretation using different algorithms exhibited distinct sensitivity and specificity in CRC detection

**Fig 1:**
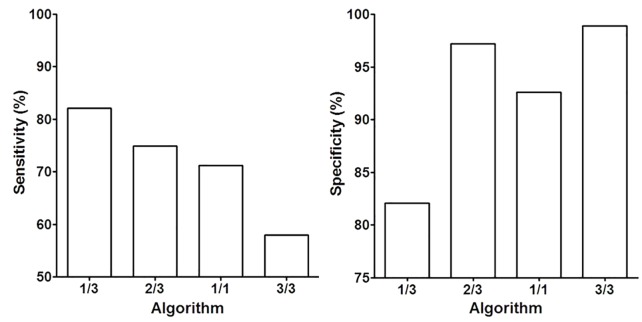
The Epi proColon 2.0 CE assay exhibited high sensitivity and specificity in CRC detection. The sensitivity and specificity of the Epi proColon 2.0 CE assay in the opportunistic screening study with various algorithm were shown. Values for sensitivity were shown on the left panel and values for specificity were shown on the right panel for 1/3, 2/3, 1/1 and 3/3 algorithm.

In order to investigate the effect of various algorithms on detection performance, the values for sensitivity and specificity were calculated and compared. It can be clearly seen from Table 2 and Fig 1 that 1/3 algorithm exhibited the highest sensitivity while 3/3 algorithm exhibited the lowest sensitivity. The sensitivity at 1/3 algorithm was significantly higher than that of the 2/3 (χ2 = 5.44, p<0.05), 1/1(χ2 = 12.13, p<0.001) and 3/3 (χ2 = 51.16, p<0.001) algorithm. In contrast, 3/3 algorithm exhibited the highest specificity while 1/3 algorithm exhibited the lowest specificity. The specificity of the 3/3 algorithm was significantly higher that of the 1/3 (χ2 = 79.12, p<0.001) and 1/1(χ2 = 22.39, p<0.001) algorithm, but not the 2/3 algorithm (χ2 = 3.26, p = 0.07). The sensitivity increased with the decrease in the required number of positive PCR reactions, while the specificity increased with the increase in required number of positive PCR reactions. These results showed a clear trend in the change of sensitivity and specificity based on the required positive reactions in data interpretation.

**Table 2:** Sensitivity and specificity for CRC using different algorithm.

	1/3	2/3	1/1	3/3
Sensitivity	82.4%	75.1%	71.3%	58.0%
	(303/369)	(277/369)	(263/369)	(214/369)
Specificity	82.0%	97.1%	92.7%	98.8%
	(402/490)	(476/490)	(454/490)	(484/490)

### Stage-dependent CRC positive detection rate is dependent on the choice of algorithm

**Fig 2:**
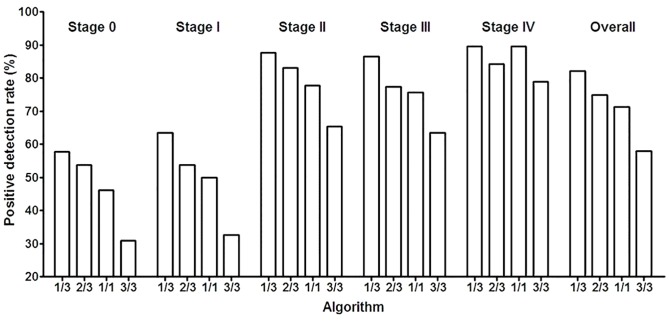
Early-stage CRC can be detected by the SEPT9 assay. The positivity detection rate was shown for each colorectal cancer stage in the opportunistic screening using various algorithm. Data was shown from stage 0 to stage IV and the overall PDR with 1/3, 2/3, 1/1 and 3/3 algorithm.

The detection of early stage CRC (stage 0 and I) is extremely important for improving the effect of early therapy and 5-year survival rate. We also further investigated the detection capability for early-stage CRC, and recruited 21 CRC subjects at stage 0 (carcinoma in situ, CIS) and 42 subjects at stage I to study the positive detection rate (PDR) of Epi proColon 2.0 CE using various algorithms. The PDR for each CRC stage exhibited the same trend as the overall sensitivity at various algorithms (Table 3 and Fig 2). Although no statistically significant differences were found between the PDR of each algorithm for stage 0, a clear trend in the PDR can be observed with the change of algorithm (Fig 2). 57.1% and 64.3% of stage 0 and stage I CRC was detected, respectively, using 1/3 algorithm, while 52.4% and 54.8% of stage 0 and I CRC can be detected with 2/3 algorithm. This result suggests that Epi proColon 2.0 can detect more than half of the CRC cases with the 1/3 or 2/3 algorithm. The PDR also exhibited a stage-dependent increase, in which higher PDR correlated with higher stage. This is consistent with previous observations with Epi proColon 2.0 CE [8].

**Table 3:** Positive detection rate for each CRC stage using different algorithm.

	1/3	2/3	1/1	3/3
Stage 0	57.1%	52.4%	47.6%	33.3%
	(12/21)	(11/21)	(10/21)	(7/21)
Stage I	64.3%	54.8%	50.0%	33.3%
	(27/42)	(23/42)	(21/42)	(14/42)
Stage II	87.6%	82.9%	78.1%	65.7%
	(92/105)	(87/105)	(82/105)	(69/105)
Stage III	87.8%	78.6%	76.3%	64.1%
	(115/131)	(103/131)	(100/131)	(84/131)
Stage IV	93.3%	86.7%	93.3%	80.0%
	(14/15)	(13/15)	(14/15)	(12/15)
Not specified	78.2%	72.7%	65.5%	50.9%
	(43/55)	(40/55)	(36/55)	(28/55)
Overall	82.4%	75.1%	71.3%	58.0%
	(303/369)	(277/369)	(263/369)	(214/369)

### Positive detection rate of GI diseases exhibited variation with the different algorithms

**Fig 3:**
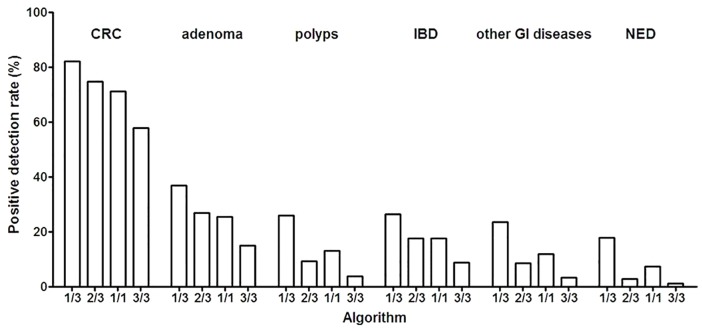
Algorithm affects the detection performance on various colorectal diseases. The positivity detection rate was shown for serveral types of colorectal diseases. in the opportunistic screening using various algorithm. Data was shown for CRC, adenoma, polyps, IBD, other GI diseases and NED with 1/3, 2/3, 1/1 and 3/3 algorithm.

Although the *SEPT9* assay is regarded as a test for CRC, it exhibited certain PDR for GI diseases other than CRC. Similar trends in PDR were found in other GI diseases as in CRC with various algorithms (Table 4 and Fig 3). Data interpretation using 1/3 algorithm detected 37.2% of patients with adenomas (χ2 = 20.04, p<0.001), showing significant difference compared with the PDR of the NED group (18.0%), and data interpretation using the 2/3 algorithm detected 26.5% of adenoma patients (χ2 = 76.19, p<0.001), 9.2% polyps (χ2 = 8.09, p<0.01) and 17.6% IBD (χ2 = 17.73, p<0.001), also showing significant difference compared with the PDR of the NED group (2.9%). A similar trend was also observed with 1/1 and 3/3 algorithm (Table 4). These results suggest that the *SEPT9* assay can distinguish between adenoma/polyps and the healthy subjects (NED) with the most commonly used algorithm (1/3 and 2/3) in data interpretation, although the PDR for them is not ideal for it to be an assay for adenoma/polyps detection.

**Table 4:** Positive detection rate for colorectal diseases using different algorithm.

	1/3	2/3	1/1	3/3
CRC	82.4%	75.1%	71.3%	58.0%
	(303/369)	(277/369)	(263/369)	(214/369)
Adenoma	37.2%	26.5%	25.7%	15.0%
	(42/113)	(30/113)	(29/113)	(17/113)
Polyps	26.4%	9.2%	12.6%	3.4%
	(23/87)	(8/87)	(11/87)	(3/87)
IBD	25.9%	18.5%	18.5%	7.4%
	(7/27)	(5/27)	(5/27)	(2/27)
Other GI Diseases	23.4%	8.5%	12.8%	3.4%
	(11/47)	(4/47)	(6/47)	(2/47)
NED	18.0%	2.9%	7.3%	1.2%
	(88/490)	(14/490)	(36/490)	(6/490)

### SensiColon, a newly optimized SEPT9 assay using a single PCR reaction, exhibited equivalent performance with Epi proColon 2.0 CE assay using the 2/3 algorithm

**Fig 4:**
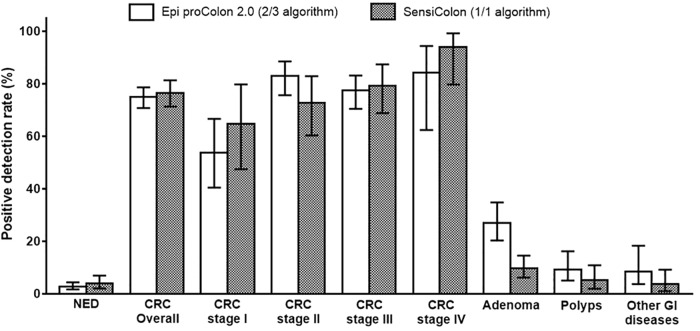
The SensiColon exhibited essentially the same performance as the Epi proColon 2.0 CE assay. Comparison of the positive detection rate was shown for Epi proColon 2.0 CE and the SensiColon assays in various colorectal diseases. 2/3 algorithm was used for data analysis in Epi proColon 2.0 CE assay, and 1/1 algorithm was used for SensiColon assay. Data was shown for PDR of all stages of CRC, adenoma, polyps, other GI diseases and NED for both assays.

**Fig 5:**
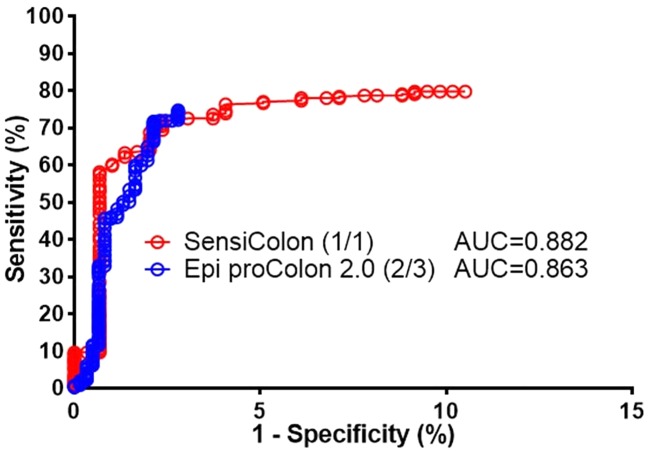
The ROC curves showed no difference in performance for the two types of SEPT9 assays. Comparison of the ROC curves was shown for Epi proColon 2.0 and SensiColon assays. 2/3 algorithm was used for data analysis in Epi proColon 2.0 CE assay, and 1/1 algorithm was used for SensiColon assay. No significant difference was found in AUC between the two assays.

We recently developed a new simplified *SEPT9* assay (SensiColon) and performed an opportunistic screening study in Chinese population [7]. Here we compared the performance of the two assays in the opportunistic screening setting. As shown in Table 5 and Fig 4, the PDR for SensiColon and Epi proColon 2.0 CE was compared in CRC, adenoma, polyps, other GI diseases and NED groups. Since the 2/3 algorithm is the recommended methods for data interpretation for Epi proColon 2.0 CE, SensiColon with a single 60 μl total PCR volume (approximately 1.8 ml plasma equivalent for each PCR reaction) was compared with Epi proColon 2.0 CE with 2/3 algorithm and 30 μl total PCR volume (approximately 0.9 ml plasma equivalent for each PCR reaction). It can be clearly seen that SensiColon exhibited an essentially identical PDR for CRC, including all stages, and in Polyps, other GI diseases and NED groups, although the PDR for Epi proColon 2.0 CE in adenoma appears to be higher than that for SensiColon. This could be due to the distinct composition of different types of adenoma, such as advanced adenoma and non-advanced adenoma, in the two opportunistic screening trials. Furthermore, the ROC curves from both assays appear to be similar to each other and the AUC from the assays is essentially identical (Fig 5). These results indicate that the newly optimized SensiColon using a single PCR exhibited equivalent performance with Epi proColon 2.0 CE assay using 2/3 algorithm.

**Table 5:** Comparison of the positive detection rate between Epi proColon 2.0 and SensiColon.

Positive Detection Rate		Epi proColon 2.0 (30 μl PCR,2/3 algorithm)	SensiColon (60 μl PCR, 1/1 algorithm)	χ^2^, p
CRC	Overall	75.1% (277/369)	76.6% (223/291)	0.22,0.64
	I	54.8% (23/42)	64.9% (24/37)	0.83,0.36
	II	82.9% (87/105)	72.7% (48/66)	2.50,0.11
	III	78.6% (103/131)	79.3% (65/82)	0.01,0.91
	IV	86.7% (13/15)	93.9% (31/33)	0.71,0.40
Adenoma		26.5% (30/113)	9.8% (21/214)	15.73,P<0.001
Polyps		9.2% (8/87)	5.2% (6/116)	1.25,0.26
Other GI Diseases		8.5% (4/47)	3.7% (4/108)	1.55,0.21
NED		2.9% (14/490)	4.1% (12/295)	0.84,0.36

## Discussion

### Pros and cons in data interpretation of multiple RT-pPCR assay with different algorithms

The multiple RT-qPCR assay has provided a strong tool for DNA methylation detection in various diseases, especially in cancer, where abnormal hypermethylation at promoter regions or around CpG islands is frequently detected. In the Epi proColon assay, abnormal hypermethylation of multiple methylation sites at the *SEPT9* gene promoter region is detected using specific probes and blockers during RT-qPCR reaction [9,10]. Parallel multiple PCR reactions can enhance the detection sensitivity at the price of increasing the false positive rate, while interpretation of PCR data using different algorithms greatly affects the sensitivity and specificity of an assay. The optimal algorithm would be the one that best balances the sensitivity and specificity. In this study, it is clear that 1/3 algorithm exhibited the highest sensitivity with the lowest specificity, and 3/3 algorithm exhibited the highest specificity with the lowest sensitivity for overall cancer detection. This is also true for the stage-related positive detection of CRC and the detection of adenoma and other GI diseases. Since the 2/3 algorithm exhibited slightly better sensitivity and specificity than the 1/1 algorithm, it is regarded as the optimal algorithm for Epi proColon 2.0 CE assay. Therefore, the 2/3 algorithm is recommended as the standard method for data interpretation in the instructions for use.

Technically, multiple PCR reactions increase the needs for sample quantity and this could be a challenge for clinical applications of RT-qPCR assays. For example, the current *SEPT9* assay requires at least 3.5 ml plasma from 10 ml whole blood to perform three parallel PCR reactions. This amount of blood is higher than most *in vitro* blood-based assays. However, detection of low concentration of abnormal methylation from circulating tumor DNA (ctDNA) in the background of much higher normal DNA requires sufficient blood, as the quantity of abnormal ctDNA in circulation is very low. Highly sensitive PCR and probes are also required for the blood-based ctDNA assay as the detection of abnormal methylation of less than 10 genome copies is common to discriminate cancer from normal subjects. In the *SEPT9* assay, the limit of detection (LOD) could be as low as 7.8 pg/mL (95% CI 6–11 pg/mL), corresponding to <2 genome copies of methylated *SEPT9* per milliliter of plasma [4]. Multiple replicate PCR reactions enable the detection of such low amount of abnormal methylation.

### The choice of algorithm in multiple RT-pPCR assay is dependent on the purpose of an assay

The multiple RT-qPCR reaction for abnormal methylation detection can be used in early cancer detection and screening. The application of the assay in early detection requires relatively high specificity as a high rate of false positive detection would lead to a high rate of costly follow-up procedures. However, high sensitivity is also important for detecting as many potential CRC patients as possible. The 2/3 algorithm therefore provided the best balance between sensitivity and specificity as it detected 3/4 of cancer patients with less than about 3% false positive rate in this study.

In contrast, in average-risk population, both CRC incidence and the detection positivity rate are low. It was reported by the PRESEPT study that the CRC incidence was 0.67% (53/7941) while the overall positivity rate for *SEPT9* assay was roughly 10% (153/1516) [11]. The clinical performance of the Epi proColon *SEPT9* assay was evaluated in 1544 subjects from the PRESEPT study. 30 out of 44 CRC patients were detected by the assay, representing a screening sensitivity of 68%, while 1182 out of 1500 non-CRC patients (including advanced adenoma, small polyps, and no evidence of diseases) were confirmed to be negative, representing an adjusted specificity of 80%. The sensitivity and specificity in this screening study were calculated using 1/3 algorithm, as sensitivity in CRC screening apparently overweighs specificity in order to identify as many potential CRC patients as possible, including those with precancerous conditions[4,11]. In a second trial comparing with the OC-Auto fecal immunochemical test (FIT), the Epi proColon test exhibited a sensitivity of 73.3% at 81.5% specificity [12]. The Epi proColon assay with 1/3 algorithm was therefore approved by the US FDA as the first blood-based CRC screening assay.

There is an argument among physicians, patients, and testing providers regarding the 2/3 algorithm. A test result at 1/3 positive should be determined as negative according to 2/3 algorithm, but patients are indeed at high risk with earlier stage of colorectal cancer or developing into colorectal cancer in foreseen future. Patients with a positive Epi proColon test result should be referred for diagnostic colonoscopy. It would be a good idea to inform physicians and patients the high risk of negative result with 1/3 positive in the test, and encourage a further colonoscopy examination for patients who do not have colonoscopy examination recently before the test. The US FDA also recommends that the Epi proColon test results should be used in combination with assessment from physicians and individual risk factors in guiding patient management. If a patient exhibits negative result in colonoscopy, more frequent colonoscopy examination is recommended. This action may lead to increased burden of public health service, but will reduce the therapeutic costs for CRC.

### Optimization of RT-pPCR assay and the algorithm facilitates CRC screening and early detection

Ideally, multiple PCRs could be replaced by single PCR to facilitate clinical application. This could reduce the amount of samples needed, reduce the time needed per run, reduce the costs per run, simplifies the test procedure, and increase the test throughput per run. These are important in a screening assay, in which fast, inexpensive, convenient and reliable tests are the key for success. We recently reported the development of a simplified new *SEPT9* assay (SensiColon) using a 60 μl single PCR reaction with a 1/1 algorithm. This new assay exhibited no difference in performance to Epi proColon 2.0 CE assay using a 30 μl PCR reaction with the 2/3 algorithm in an opportunistic screening setting, except that the PDR for adenoma in Epi proColon 2.0 CE was higher than that in the new assay (Figs 4 and 5 and Table 5). This could be due to the composition of adenomas in the different populations, as the PDR for advanced adenomas (AA) was shown to be higher than that of the overall adenoma [8].

Since most cycle threshold (Ct) values from normal controls were not detected in the PCR reaction, we had to set the Ct values to 45 (the maximal number of PCR cycles we ran in the assay) for those undetected normal controls to plot the curve. This limitation led to the lack of specificity data points for Ct values >45. Therefore, no data were plotted above certain percentage for 1-specificity (the X-axis) in the ROC curves for both Epi proColon 2.0 CE and SensiColon assays. The fact that the two ROC curves exhibited similar shape and AUC values also suggests that the performance of the two assays in opportunistic screening is identical.

The simplified new *SEPT9* assay reduced the required quantity of blood samples and the amount of DNA by 1/4 to 1/3 without compromising the test performance. It also increased the PCR throughput three times and reduced the cost of the assay, facilitating its application in large-scale screening. In addition, a single PCR reaction is easier to manipulate and interpret and reduces the chance of errors. The simplified assay also expands the options for applicable PCR machines to ABI 7500 and other PCR equipment, not confining to ABI 7500 fast, fast DX, and Roche 480 I/II.

While an increase in the PCR reaction volume may increase the non-specific signal and reduce the specificity, this can be overcome by adjusting the cutoff value. In the new *SEPT9* assay, we adjusted the cutoff value to 41, instead of 45 as in Epi proColon 2.0 CE, and achieved the same specificity and maintained the same sensitivity as Epi proColon 2.0 CE assay. The detailed optimization procedure was outlined in our previous publication^7^. This is also true if the sensitivity of SensiColon is adjusted to the identical value of 82.4% as Epi proColon 2.0 CE at 1/3 algorithm. The specificity of SensiColon at 82.4% sensitivity is calculated to be 81.1% from the ROC curve, which is very similar to that of the Epi proColon 2.0 CE (82.0% specificity). This result again proves that SensiColon has essentially the same performance as Epi proColon 2.0 CE. On the other hand, the sensitivity and specificity of an assay is partially dependent on the intrinsic properties of a marker, especially when the detection capability is pushed to its limit. Further enhancement of detection sensitivity and specificity may not lead to improvement of clinical performance, but can reduce the amount of clinical samples required in an assay. This was illustrated during the development of the new *SEPT9* assay. The enhancement of specificity allows optimized discrimination between normal and abnormal clinical samples, which is one of the reasons that a single *SEPT9* methylation marker exhibited much higher sensitivity in CRC detection than other methylation and protein markers. The methods used in the new *SEPT9* assay optimization can be used in optimizing other PCR assays aiming at detection of tiny amount of templates.

The current *SEPT9* assays, including the Epi proColon 2.0 CE and the SensiColon, can be further optimized by reducing the amount of blood needed for CRC detection. This could be a key step to enhance the compliance in countries where 10 ml blood draw for a single assay is not common. Table 6 shows the volume of blood, plasma, DNA elution and PCR reaction in both assays, with corresponding actual and predictive sensitivity and specificity. The Epi proColon 2.0 CE assay currently uses 3.5 ml plasma from 10 ml blood, and the elution volume is 60 μl with 45 μl used in three PCR reactions (equivalent to 2.7 ml plasma). It showed a sensitivity of 75.1% with a specificity of 97.1% at the current setting. In contrast, SensiColon collect the same amount of blood and plasma and uses the same volume of elution, while only uses half of the elution (equivalent to 1.8 ml plasma) in a single PCR reaction. It showed a sensitivity of 76.6% with a specificity of 95.9%. If the amount of blood is reduced to 5 ml for Epi proColon 2.0 CE assay, the equivalent plasma used in the assay would be 1.8 ml, which is identical to that of the current SensiColon assay, and it can be predicted that its performance would be similar to that of the SensiColon if a single PCR is performed. Similarly, if the amount of blood is further reduced to 3 ml in SensiColon assay, the equivalent plasma would be 1 ml, and the performance would be similar to that of the Epi proColon 2.0 CE assay with 1/1 algorithm (Table 2). Therefore, it is possible to reduce the blood volume to 3 ml without substantial compromise in detection sensitivity and specificity. Further validation experiments are needed to prove the prediction.

**Table 6:** The SEPT9 assay performance prediction based on equivalent plasma volume in PCR reaction.

	blood volume	plasma volume	DNA elution volume	Volume used in PCR	plasma equivalent	sensitivity	specificity	note
Epi proColon 2.0 CE	10 ml	3.5 ml	60 μl	45 μl	2.7 ml	75.1%	97.1%	actual data
	5 ml	1.8 ml	30 μl	30 μl	1.8 ml	76.6%	95.9%	predictive data
SensiColon	10 ml	3.5 ml	60 μl	30 μl	1.8 ml	76.6%	95.9%	actual data
	3 ml	1.0 ml	15 μl	15 μl	1.0 ml	71.3%	92.7%	predictive data

## Conclusions

The selection of algorithm in a multiple PCR assay is crucial for test performance. The optimal algorithm would be the one that best balances sensitivity and specificity. The application of algorithm is dependent on the purpose of an assay. Screening for potential high-risk population normally needs high sensitivity while tests aiming at early detection normally require high specificity to avoid costly follow-up procedures. PCR assays with plasma samples can be optimized by increasing the equivalent plasma volume or reducing the number of reactions needed, which can be achieved together to facilitate its clinical application. Further optimization is worthwhile to make cancer liquid biopsy a routine assay for potential patients.

## Supporting Information

S1 Dataset (ZIP)

## References

[1] Schreuders EH, Ruco A, Rabeneck L, Schoen RE, Sung JJ, Young GP, et al Colorectal cancer screening: a global overview of existing programmes. Gut. 2015; 64:1637–1649. 10.1136/gutjnl-2014-309086 26041752

[2] American Cancer Society: Colorectal Cancer Facts & Figures 2011e2013. Atlanta, American Cancer Society, 2011.

[3] deVos T, Tetzner R, Model F, Weiss G, Schuster M, Distler J, et al Circulating methylated *SEPT9* DNA in plasma is a biomarker for colorectal cancer. Clin Chem. 2009; 55:1337–1346. 10.1373/clinchem.2008.115808 19406918

[4] Potter NT, Hurban P, White MN, Whitlock KD, Lofton-Day CE, Tetzner R, et al Validation of a real-time PCR-based qualitative assay for the detection of methylated *SEPT9* DNA in human plasma. Clin Chem. 2014; 60:1183–1191. 10.1373/clinchem.2013.221044 24938752

[5] Li Y, Song L, Gong Y, He B. Detection of colorectal cancer by DNA methylation biomarker *SEPT9*: past, present and future. Biomark Med. 2014; 8:755–769. 10.2217/bmm.14.8 25123042

[6] Song L, Li Y. *SEPT9*: a specific circulating biomarker for colorectal cancer. Adv Clin Chem. 2015; 72:171–204. 10.1016/bs.acc.2015.07.004 26471083

[7] Wu D, Zhou G, Jin P, Zhu J, Li S, Wu Q, et al Detection of Colorectal Cancer Using a Simplified *SEPT9* Gene Methylation Assay Is a Reliable Method for Opportunistic Screening. J Mol Diagn. 2016 4 28. 10.1016/j.jmoldx.2016.02.005 27133379

[8] Jin P, Kang Q, Wang X, Yang L, Yu Y, Li N, et al Performance of a secondgeneration methylated *SEPT9* test in detecting colorectal neoplasm. J Gastroenterol Hepatol. 2015; 30:830–833. 10.1111/jgh.12855 25471329

[9] Lofton-Day C, Model F, Devos T, Tetzner R, Distler J, Schuster M, et al DNA methylation biomarkers for bloodbased colorectal cancer screening. Clin Chem. 2008; 54:414–423. 10.1373/clinchem.2007.095992 18089654

[10] Grützmann R, Molnar B, Pilarsky C, Habermann JK, Schlag PM, Saeger HD, et al Sensitive detection of colorectal cancer in peripheral blood by septin 9 DNA methylation assay. PLoS One. 2008; 3:e3759 10.1371/journal.pone.0003759 19018278 PMC2582436

[11] Church TR, Wandell M, Lofton-Day C, Mongin SJ, Burger M, Payne SR, et al Prospective evaluation of methylated *SEPT9* in plasma for detection of asymptomatic colorectal cancer. Gut. 2014; 63:317–325. 10.1136/gutjnl-2012-304149 23408352 PMC3913123

[12] Johnson DA, Barclay RL, Mergener K, Weiss G, König T, Beck J, et al Plasma Septin9 versus fecal immunochemical testing for colorectal cancer screening: a prospective multicenter study. PLoS One. 2014; 9:e98238 10.1371/journal.pone.0098238 24901436 PMC4046970

