# Supplementary material for: Algorithm Optimization in Methylation Detection with Multiple RT-qPCR
Source: PLoS One. 2016 Nov 29;11(11):e0163333. doi: 10.1371/journal.pone.0163333 (PMC5127507; doi:10.1371/journal.pone.0163333)
Supplement: S1 Dataset — (ZIP) [file pone.0163333.s001.zip › Minimal dataset revised/Figure captions and legends.docx]

Figure 1. **The Epi proColon 2.0 CE assay exhibited high sensitivity and specificity in CRC detection.** The sensitivity and specificity of the Epi proColon 2.0 CE assay in the opportunistic screening study with various algorithm were shown. Values for sensitivity were shown on the left panel and values for specificity were shown on the right panel for 1/3, 2/3, 1/1 and 3/3 algorithm.

Figure 2. **Early-stage CRC can be detected by the SEPT9 assay.** The positivity detection rate was shown for each colorectal cancer stage in the opportunistic screening using various algorithm. Data was shown from stage 0 to stage IV and the overall PDR with 1/3, 2/3, 1/1 and 3/3 algorithm.

Figure 3．**Algorithm affects the detection performance on various colorectal diseases.** The positivity detection rate was shown for serveral types of colorectal diseases. in the opportunistic screening using various algorithm. Data was shown for CRC, adenoma, polyps, IBD, other GI diseases and NED with 1/3, 2/3, 1/1 and 3/3 algorithm.

Figure 4．**The SensiColon exhibited essentially the same performance as the Epi proColon 2.0 CE assay.** Comparison of the positive detection rate was shown for Epi proColon 2.0 CE and the SensiColon assays in various colorectal diseases. 2/3 algorithm was used for data analysis in Epi proColon 2.0 CE assay, and 1/1 algorithm was used for SensiColon assay. Data was shown for PDR of all stages of CRC, adenoma, polyps, other GI diseases and NED for both assays.

Figure 5．**The ROC curves showed no difference in performance for the two types of SEPT9 assays.** Comparison of the ROC curves was shown for Epi proColon 2.0 and SensiColon assays. 2/3 algorithm was used for data analysis in Epi proColon 2.0 CE assay, and 1/1 algorithm was used for SensiColon assay. No significant difference was found in AUC between the two assays.
