# Supplementary figures and images for: Algorithm Optimization in Methylation Detection with Multiple RT-qPCR
Source: PLoS One. 2016 Nov 29;11(11):e0163333. doi: 10.1371/journal.pone.0163333 (PMC5127507; doi:10.1371/journal.pone.0163333)

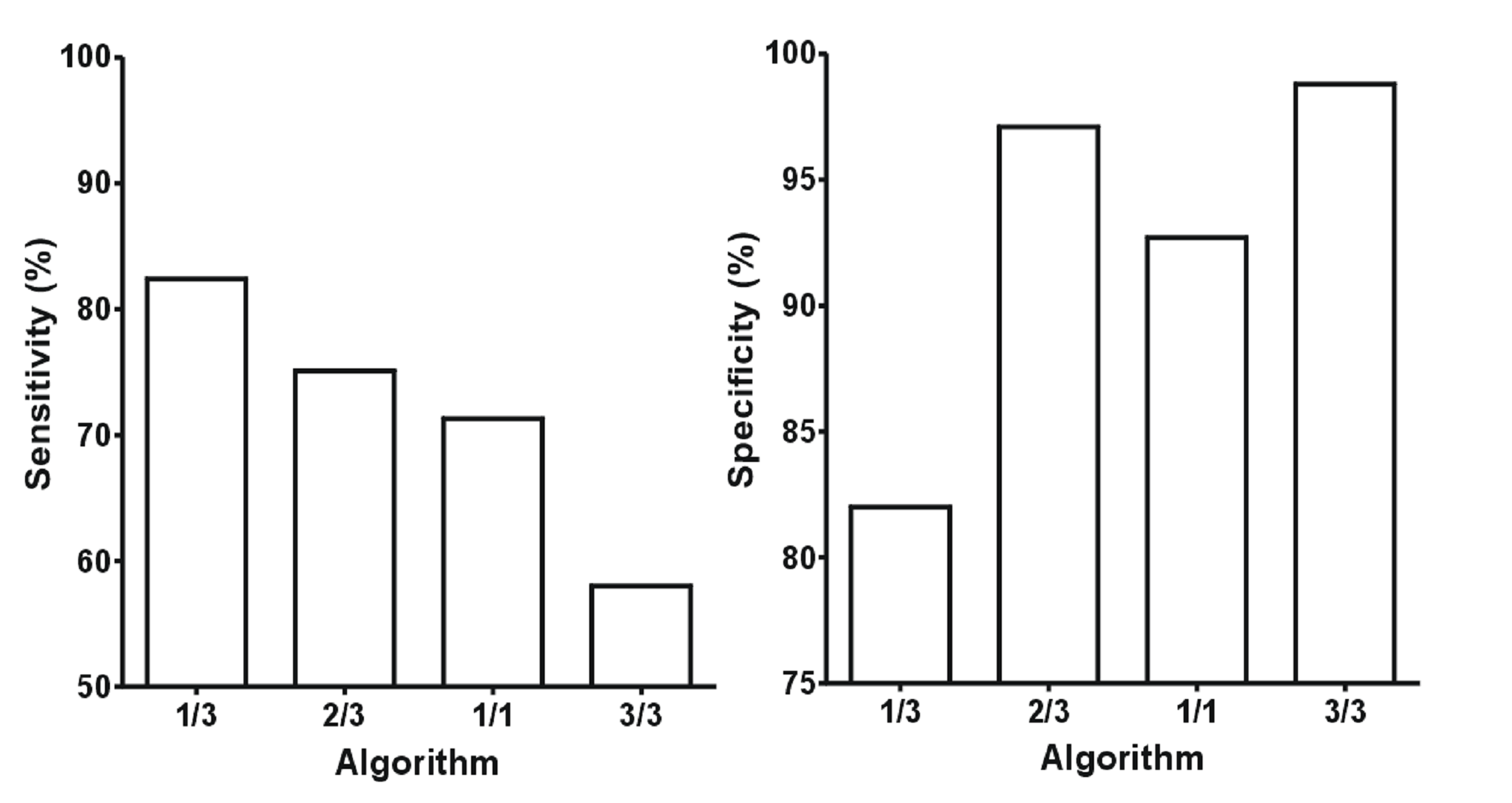

Supplement: S1 Dataset — (ZIP) [file pone.0163333.s001.zip › Minimal dataset revised/Fig1.TIF]

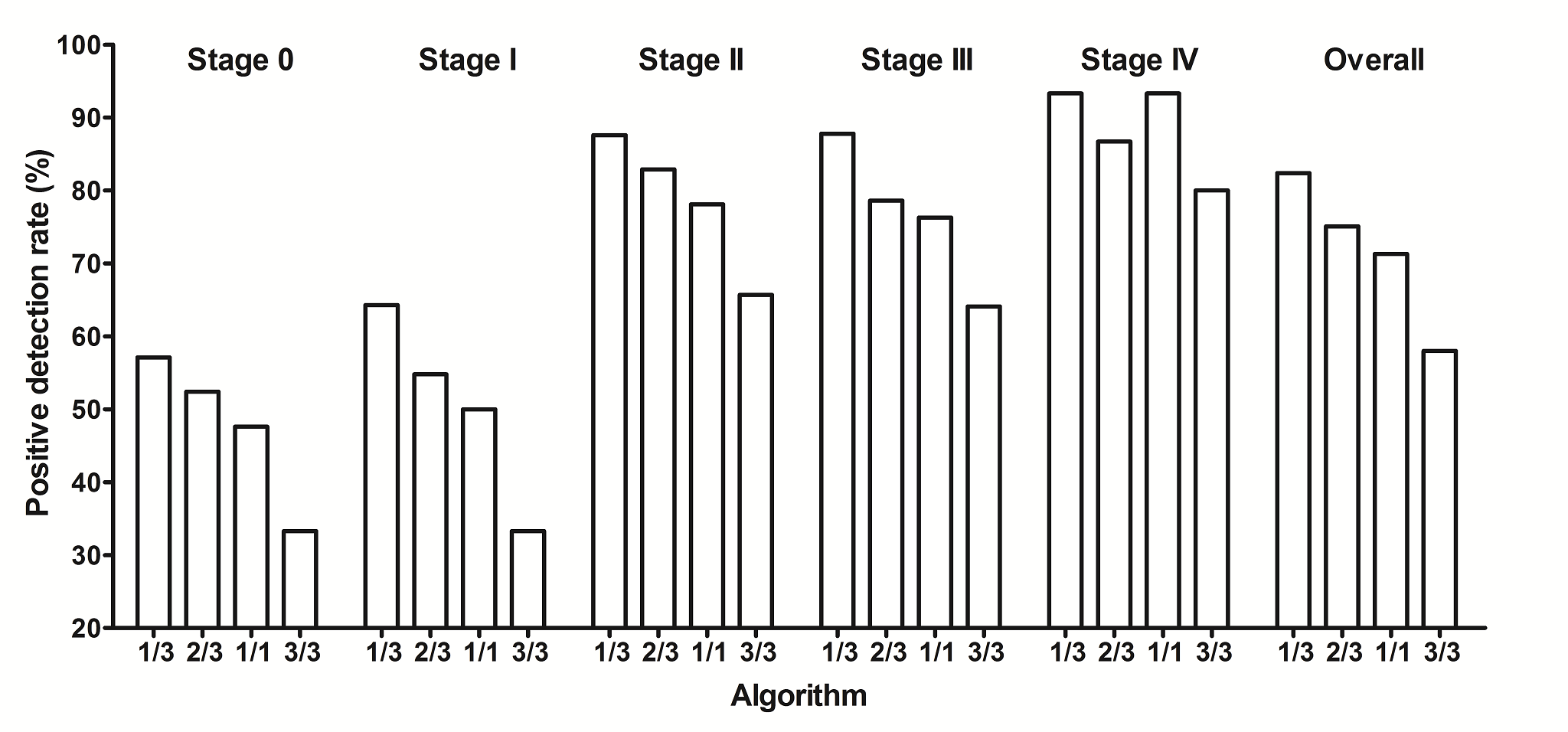

Supplement: S1 Dataset — (ZIP) [file pone.0163333.s001.zip › Minimal dataset revised/Fig2.tif]

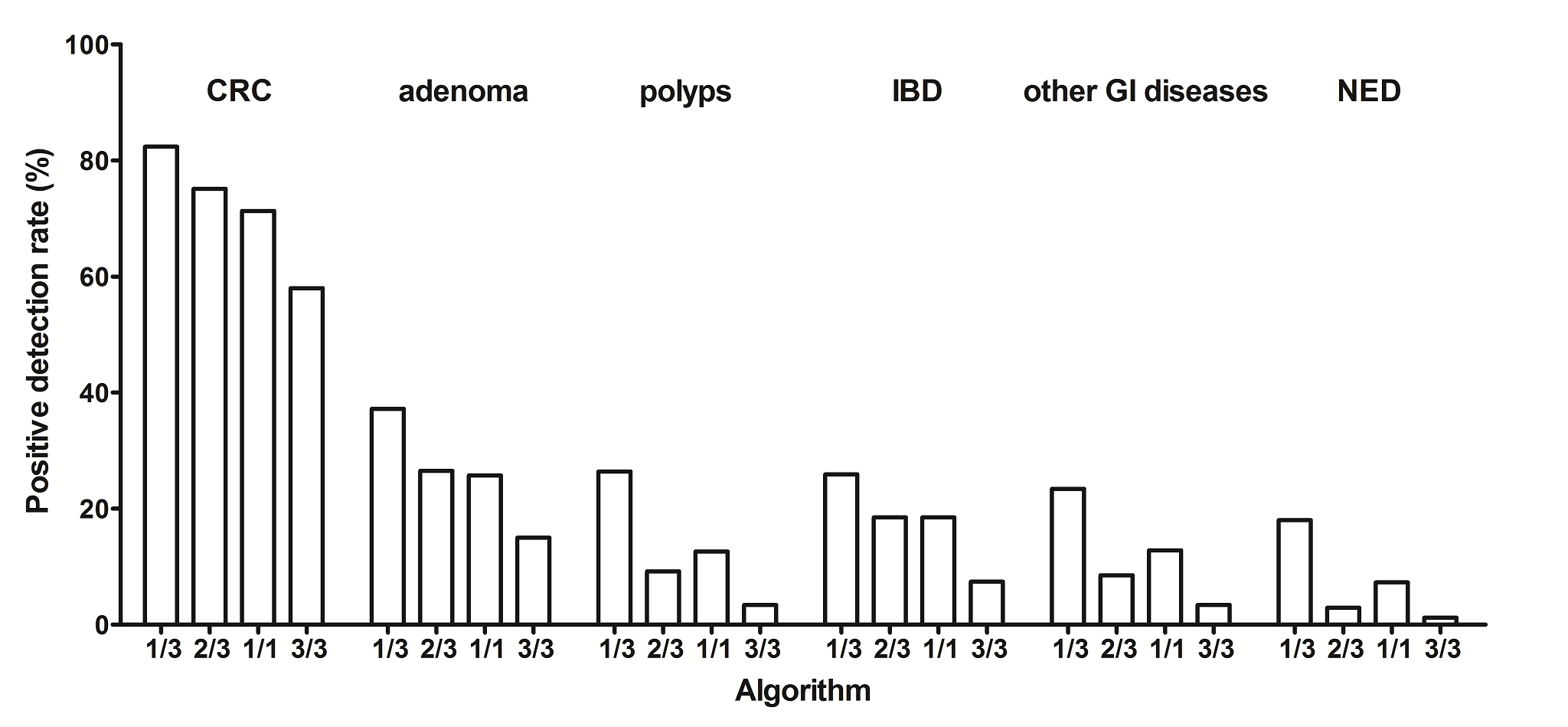

Supplement: S1 Dataset — (ZIP) [file pone.0163333.s001.zip › Minimal dataset revised/Fig3.tif]

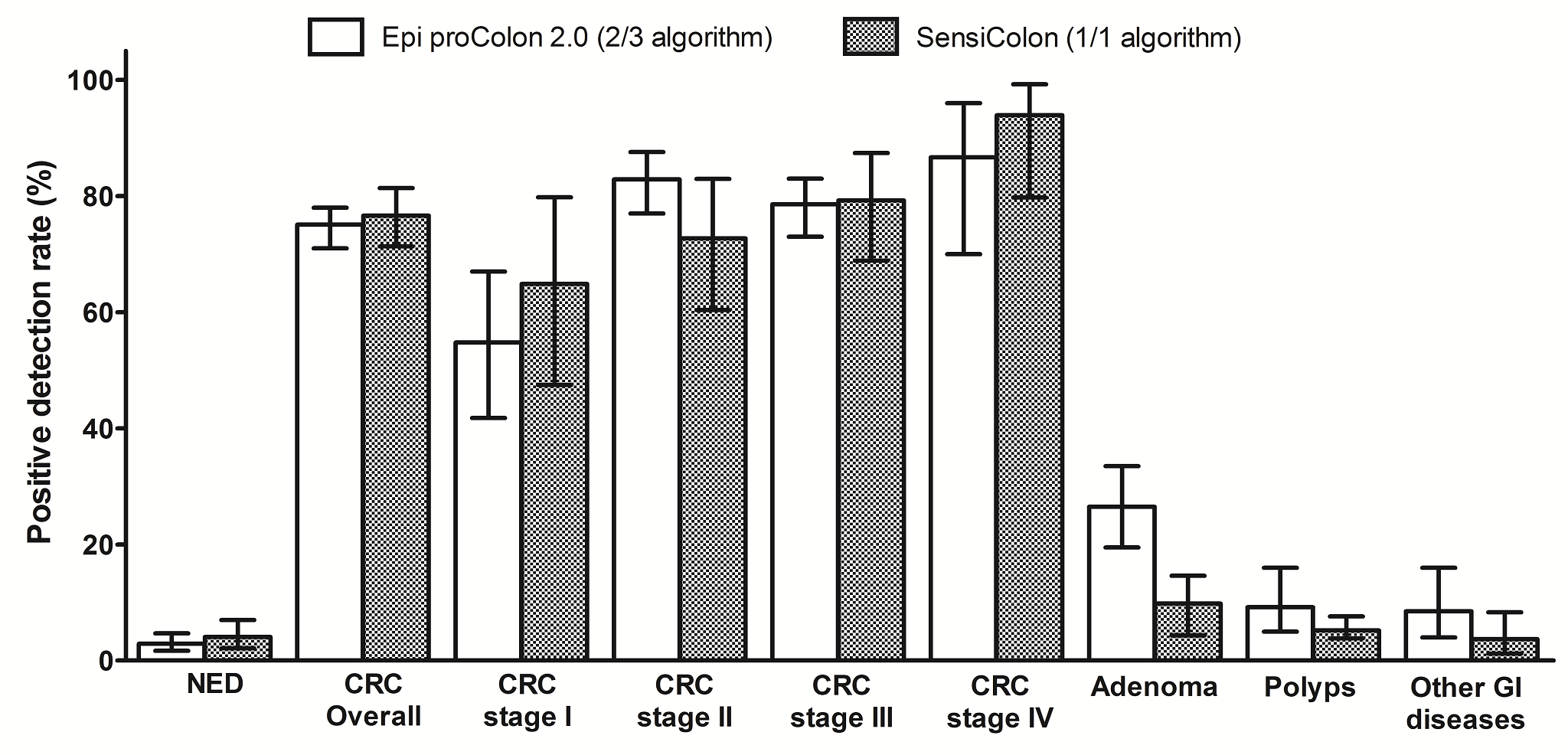

Supplement: S1 Dataset — (ZIP) [file pone.0163333.s001.zip › Minimal dataset revised/Fig4.tif]

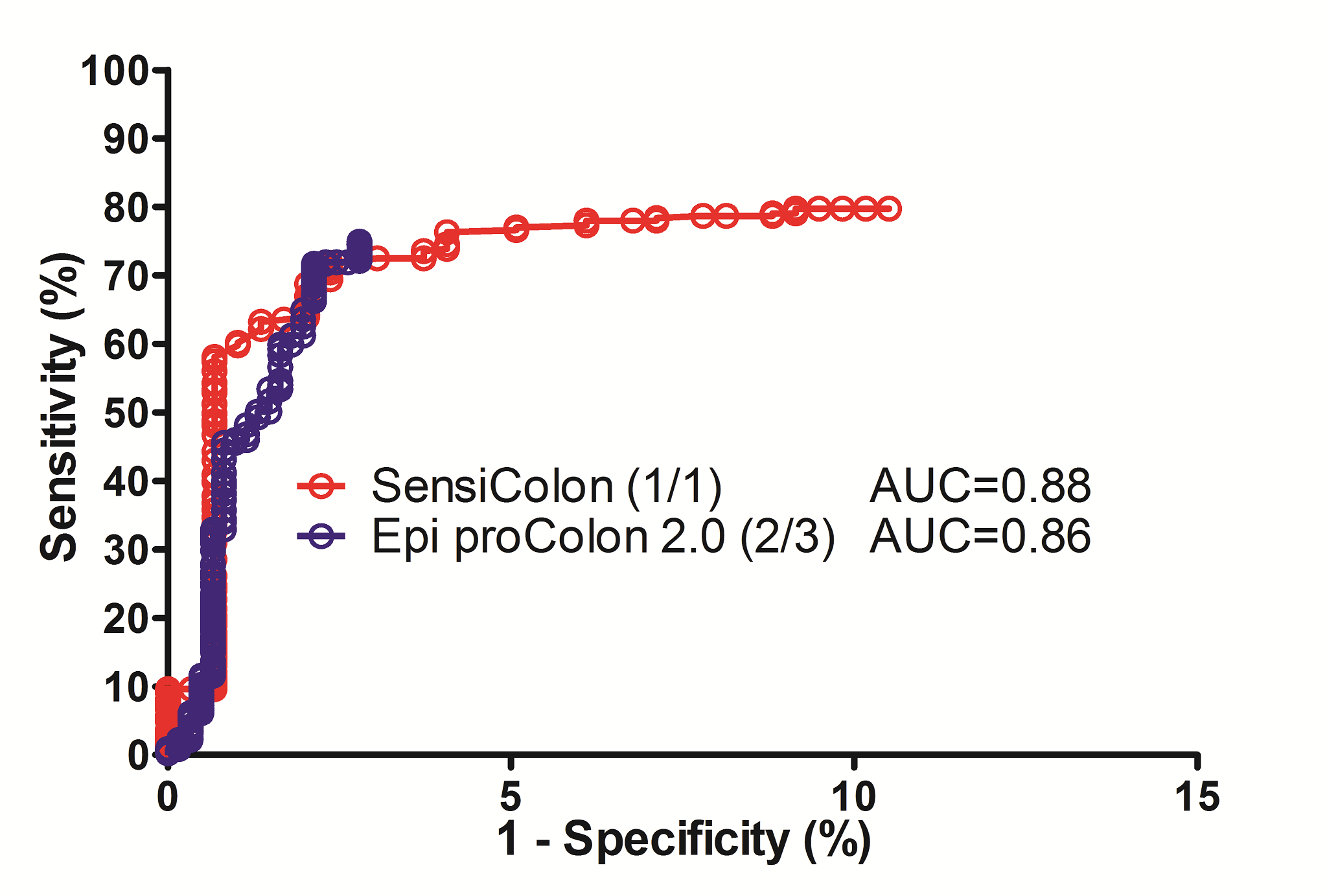

Supplement: S1 Dataset — (ZIP) [file pone.0163333.s001.zip › Minimal dataset revised/FIg5.tif]
